# COVID‐19 disease severity assessment using CNN model

**DOI:** 10.1049/ipr2.12153

**Published:** 2021-03-07

**Authors:** Emrah Irmak

**Affiliations:** ^1^ Electrical‐Electronics Engineering Department Alanya Alaaddin Keykubat University Alanya Antalya Turkey

## Abstract

Due to the highly infectious nature of the novel coronavirus (COVID‐19) disease, excessive number of patients waits in the line for chest X‐ray examination, which overloads the clinicians and radiologists and negatively affects the patient's treatment, prognosis and control of the pandemic. Now that the clinical facilities such as the intensive care units and the mechanical ventilators are very limited in the face of this highly contagious disease, it becomes quite important to classify the patients according to their severity levels. This paper presents a novel implementation of convolutional neural network (CNN) approach for COVID‐19 disease severity classification (assessment). An automated CNN model is designed and proposed to divide COVID‐19 patients into four severity classes as mild, moderate, severe, and critical with an average accuracy of 95.52% using chest X‐ray images as input. Experimental results on a sufficiently large number of chest X‐ray images demonstrate the effectiveness of CNN model produced with the proposed framework. To the best of the author's knowledge, this is the first COVID‐19 disease severity assessment study with four stages (mild vs. moderate vs. severe vs. critical) using a sufficiently large number of X‐ray images dataset and CNN whose almost all hyper‐parameters are automatically tuned by the grid search optimiser.

## 1 INTRODUCTION

On the anniversary of the novel coronavirus (COVID‐19) disease, the whole world struggles with the second wave of this infectious disease; on the other hand, all countries try to prepare for the third wave of the disease, which will probably be even more severe according to the scientists. Based on the official data reported over the past year, the number of people infected and died of COVID‐19 has exceeded 77 and 1.7 million, respectively [1]. It seems likely that these numbers will increase even more according to the World Health Organisation who declared this highly infectious disease as a pandemic on 11 March 2020 [2]. All humanity, who hopes for the good news that may come from vaccine studies with clinical trials in more than one country, is also very uneasy due to the new type of mutated virus seen in more than one country, especially the United Kingdom. Although the mutant virus has been reported to be approximately 70% more contagious, it is not yet clear whether the virus is more deadly and will be resistant to vaccines to be produced or not [3]. Fever, cough, headache, fatigue, shortness of breath, dyspnea, hypoxemia, anorexia, diarrhea, muscle soreness, shivering, vomiting, expectoration, chest tightness, and abdominal pain are found to be the common clinical symptoms of COVID‐19 disease [4].

Identifying the COVID‐19 patients who need intensive clinical care using automated severity assessment methods by deep learning becomes very urgent within the pandemic. Timely assessment of COVID‐19 patients at an early stage is now an urgent task if disease progression, triage time and mortality rate are desired to be minimised. Nevertheless, accurately staging the disease severity from radiographic images is very challenging. Some researchers found that most of the COVID‐19 patients have non‐severe (mild or moderate) symptoms [5]. There are recent studies that demonstrated that the mortality rate of non‐severe COVID‐19 patients is much higher (approximately 20 times) than of severe ones [6]. Another study showed that early identification of COVID‐19 patients that can progress to the severity and critical stages is crucial now that the average time from the first symptom to shortness of breath is only five days and to acute respiratory distress syndrome is only eight days [7]. Besides, patient management and treatment type are highly dependent on the severity of the disease. For example, antivirals and oxygen therapy are given to mild or moderate COVID‐19 patients, whereas patients from severe or critical findings need intensive care units or ventilator support [8]. Chest X‐ray has been found as an important tool to distinguish lung changes as well as abnormalities due to COVID‐19 disease. Previous studies have proved that the changes in the lungs due to the COVID‐19 disease are ground‐glass opacity, crazy paving pattern, consolidation, vascular enlargement, lower lobe involvement and bilateral infiltration [9]. Researches have demonstrated that severity assessment from chest X‐ray images of COVID‐19 patients is a powerful technique that helps in fighting this highly contagious disease.

The objective and motivation of this study are to designate a fully automatic convolutional neural network (CNN) model for multi‐classification of the COVID‐19 disease severity using large publicly available datasets. To the best of the author's knowledge, this is the first attempt of COVID‐19 disease severity classification (mild vs. moderate vs. severe vs. critical) from the largest publicly available chest X‐rays datasets until the writing of this paper, using CNN whose almost all hyper‐parameters are automatically tuned by the grid search optimiser. The rest of this paper is organised as follows. Section 2 reviews related work. Section 3 introduces the datasets and methods in details. Experimental results, optimisation algorithm details are presented in Section 4. Discussions including a comparison of the proposed study with the state‐of‐the‐art studies are presented in Section 5. Section 6 concludes the paper.

## 2 RELATED WORK

### 2.1 COVID‐19 disease detection

Despite many scientific papers published about COVID‐19 using deep learning over the past year, most of them are about COVID‐19 disease detection rather than severity assessment [10, 11, 12, 13]. For example, there are several studies that made use of the lung/lobe segmentation idea for diagnosis purposes. For instance, Shan et al. [7] quantified the lung abnormalities of COVID‐19 patients by first segmenting the infection regions in the lung lobes. Another researcher proposed a deep learning‐based segmentation method to automatically stage the COVID‐19 disease severity from ground‐glass opacity and consolidation in the lung. Amyar et al. [9] introduced a multi‐task learning to segment lesions that helped assessing the COVID‐19 severity. Lung segmentation and disease severity staging were treated as two separate processes in most of these studies. That is why He et al. [12] claimed that jointly performing lung segmentation and severity assessment could accelerate the staging process and provide rich information. However, due to the low sensitivity of COVID‐19 disease detection using deep learning, the gold standard for detecting COVID‐19 is still based on reverse‐transcription polymerase chain reaction tests of swabs from the nose and throat [14]. The COVID‐19 disease severity assessment from chest X‐rays using deep learning is found to be more suited than disease detection.

### 2.2 COVID‐19 disease severity classification

Deep learning‐based disease severity assessment is more objective and quantitative in comparison to radiologist assessment, which is qualitative reports. Despite highly promising findings of deep learning methods for COVID‐19 disease severity assessment on chest X‐ray images, just a few studies related to this subject have been reported. For instance, He et al. [12] suggested a synergistic learning approach to divide disease severity into severe or non‐severe, formulating the severity assessment into a binary classification task. They obtained 98.5% accuracy using 666 chest Computed Tomography (CT) images. Zhu et al. [14] made use of the transfer learning concept with 131 chest X‐ray images from 84 patients to classify COVID‐19 patients into four stages: Mild, moderate, severe and critical. Although multi‐class severity assessment was achieved with the top model giving a mean absolute error of 8.5%, the dataset is very small to comprehensively test the CNN outcome. Moreover, they did not use the receiver operating curve (ROC) analysis or accuracy performance evaluation metrics. Li et al. [15] classified disease severity as severe and non‐severe from 531 thick‐section CT scans using an automated deep learning method. They used two imaging biomarkers: Infection portion and average infection, for severity criteria assessment and obtained an area under the curve (AUC) value of 0.97. Tang et al. [16] showed that machine learning methods based on quantitative features acquired from CT lung images can distinguish between severe and non‐severe COVID‐19 patients. The overall accuracy obtained for this binary classification was 87.5% using 176 CT images of COVID‐19 patients in total. Xiao et al. [17] developed a deep learning method based on residual CNN (ResNet34) to estimate COVID‐19 disease severity and further estimate disease progression in COVID‐19 patients. They achieved an overall accuracy of 81.9% using chest CT images of 408 COVID‐19 patients. Yu et al. [18] used a pre‐trained deep neural network to classify disease severity as severe and non‐severe using 729 CT scans of COVID‐19 patients. They achieved an overall accuracy of 95.34%. Carvalho et al. [19] exploited artificial neural network (ANN) computer‐aided diagnosis to classify COVID‐19 patients into mild, moderate and severe cases with an overall accuracy of 82% using 229 CT scans of COVID‐19 patients. Zhang et al. [20] suggested a previously developed deep learning method for COVID‐19 severity classification as mild versus moderate versus severe. The overall accuracy they obtained was 91.6% using 661 CT scans.

## 3 THE PROPOSED METHOD

### 3.1 Datasets

Finding a research dataset to be used for COVID‐19 disease researches is very challenging now that this disease is a quite newly emerged type of coronaviruses. Despite the lack of COVID‐19 data in the literature, nine datasets from different publicly available sources are carefully collected and used in this study. The first dataset is called the COVID‐19 Image Data Collection by Cohen et al. [21] and includes 930 frontal chest X‐ray images. This is a public open dataset of chest X‐ray images of patients who are positive or suspected of COVID‐19 or other viral and bacterial pneumonias. The second dataset used in this study is the COVID‐19 Radiography Database created by a researcher team from Qatar University [22]. This is a database of chest X‐ray images for COVID‐19 positive cases along with normal and viral pneumonia images. One thousand one hundred and forty‐three COVID‐19 positive images are currently available. The third dataset is by Haghanifar et al. [23] and contains 820 COVID‐19 chest X‐ray images. The fourth dataset is collected from the Italian Society of Medical and Interventional Radiology COVID‐19 database [24]. It contains real COVID‐19 disease patients’ chest X‐ray images as well as lung CT images and aims to encourage the progression of diagnostic imaging by promoting studies and research. The fifth dataset is from an image‐based social forum resource library, which is a compilation of COVID‐19 cases and resources [25]. The sixth dataset is from a Twitter thread of a cardiothoracic radiologist who has shared high‐quality positive COVID‐19 subjects [26]. The seventh dataset is by Winther et al. [27] and known as COVID‐19 Image Repository, which is an anonymised dataset of COVID‐19 cases with a focus on radiological imaging and contains 243 images. The eighth dataset is called Novel Corona Virus 2019 Dataset, which is daily level information on COVID‐19 affected cases [28]. The last dataset used in this study is COVID‐19 Open Research Dataset [29]. This dataset has been prepared by the White House and a coalition of leading groups and represents the most extensive machine‐readable coronavirus literature collection available for data mining to date.

**FIGURE 1:**
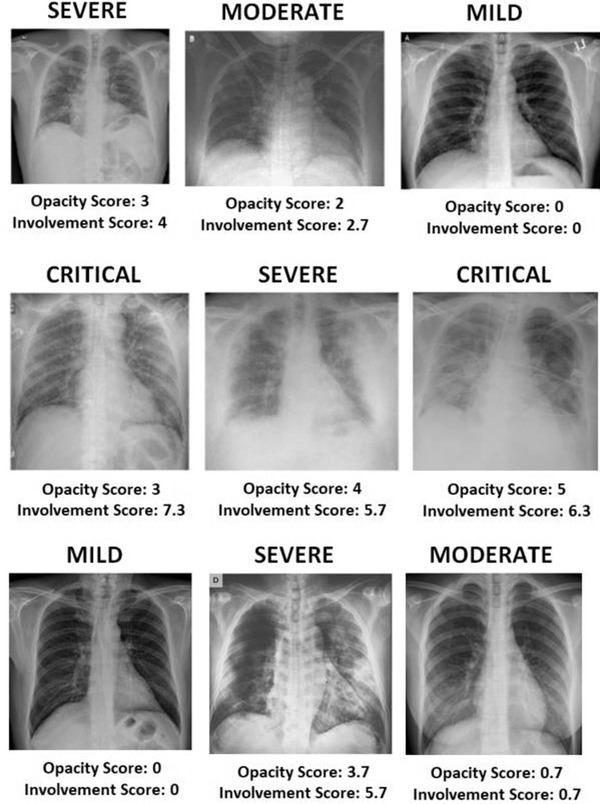
Some sample X‐ray images of COVID‐19 patients with relevant labels and disease severity scores on them. Severity scores are obtained by summing up both lung scores computed based on ground‐glass opacity and lung involvement

High accuracies from training and validation phases are not meaningful without testing the trained and hyper‐parameter‐tuned CNN on predicting unseen samples. Therefore, a test dataset is randomly assigned and separated along with training and validation datasets to test the performance of trained CNN on predicting samples; otherwise, the high accuracy may be due to biased dataset assignment (e.g. obvious images with strong characteristics from severe COVID‐19 patients). All the datasets used in this study are publicly available, and the corresponding websites are given in the reference section of this paper. It is planned to collect the largest possible number of publicly available X‐ray images of COVID‐19 that exist in the literature until the writing of this paper. Figure 1 shows some of the chest X‐ray images of COVID‐19 patients with different severity classes from the datastore. A total of 3260 X‐ray images of COVID‐19 patients are collected and used for this research. All the images used in this study are colour (jpg format) and are re‐sized to 227 × 227 × 3. For the classification task, as the study has more than 3000 samples, there are enough images to be randomly separated as training, validation and test sets having the ratio of 60:20:20 as shown in Table 1. As can be seen from Table 1, 1956 images are separated for training, 652 images are separated for validation and 652 images are separated for testing purposes, which makes 3260 images in total.

**TABLE 1:** Learning scheme of the proposed convolutional neural network (CNN) model

	**Number of images**				
**Classification groups**	**Each group**	**Total**	**Training set (60%)**	**Validation set (20%)**	**Test set (20%)**
*Mild*	1000	3260	1956	652	652
*Moderate*	950				
*Critical*	600				
*Severe*	710				

### 3.2 Experimental set‐up

The experiments of this study are performed on an NVIDIA GeForce GTX‐850 GM107 with 16 GB RAM and Intel Core i7 GPU 2.6 GHz, whereas the software environment consists of Windows 10 and MATLAB R2019a. The time spent to train the deep learning model (2608 images) was 12 min.

### 3.3 COVID‐19 lung disease severity scoring

Staging lung diseases severity using radiologist scoring is commonly the standard approach used for lung diseases severity assessments [8]. Not only the 2019‐nCoV (trial version 7) guidelines introduced by the National Health Commission of China [38] but also current COVID‐19 literature accepts that the opacity and lung disease involvement give valid results regarding COVID‐19 disease severity [15, 16]. In this study, the COVID‐19 disease severity score based on opacity degree and the lung involvement is established from chest X‐ray images of the COVID‐19 patients by two radiologists with at least 10 years of experience and blinded to each other and clinical data. The COVID‐19 lung disease severity score system, which is built upon the degree of opacity and extent of lung involvement, has been adapted from Wong et al. [30] and can be summarised as follows:

1. Opacity degree has been computed between 0 and 3 for each lung separately (left lung and right lung) and assigned as 0 if there is no opacity, 1 if ground‐glass opacity exists, 2 if there is consolidation in the lung and 3 if white‐out exists in the lung.
2. Lung involvement score is computed between 0 and 4 for each lung based on ground‐glass opacity and consolidation and assigned as 0 if there is no involvement, 1 if 0%–25% involvement exists, 2 if 25%–50% involvement exists, 3 if 50%–75% involvement exists and 4 if 75%–100% involvement exists.

The total lung severity score has been computed between 0 and 14 by summing up the opacity (0–6) and involvement scores (0–8), which is computed from each lung separately and added together. Figure 1 shows some of the chest X‐ray images severity scores including opacity and involvement scores.

### 3.4 CNN

CNN approach is one of the most popular and efficient type of deep learning methods. Unlike ANN, which uses matrix multiplication, at least one of the CNN layers uses convolution (circulation of filters for feature extraction on input) instead of matrix multiplication. CNNs generally consist of multiple trainable layers placed one after the other. Convolutional and pooling layers are the leading layers of CNN architectures, whereas fully connected (FC) and classification layers are the final stage layers. The training process is started by performing layer‐by‐layer operations just after CNN receives the input data. Finally, a final output is given to compare with the correct (actual) result. The differences between the estimated and the actual results produce an error, which is transferred to all weights by the backpropagation algorithm. Weights are updated with each iteration to reduce the error.

**FIGURE 2:**
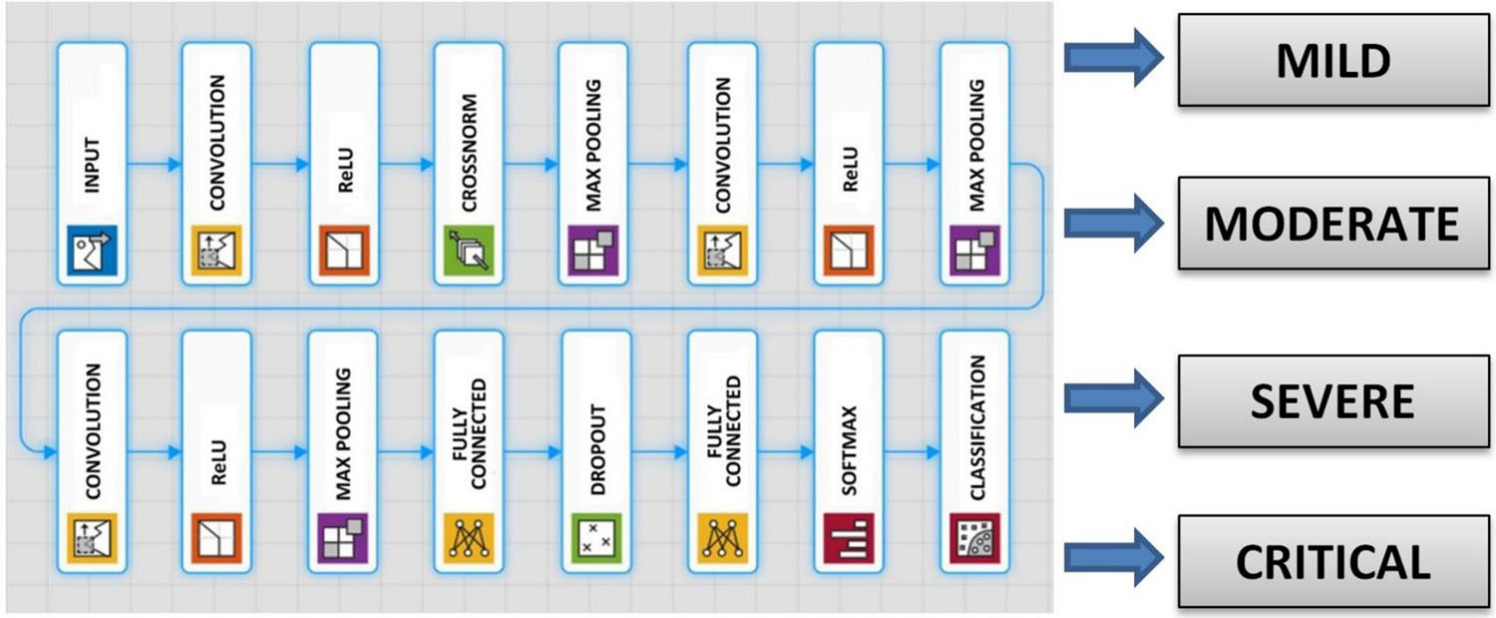
Architecture of the proposed convolutional neural network (CNN) model

This study aims to design and propose a fully automatic CNN model using large clinical chest X‐ray images for COVID‐19 disease severity assessment. Grid search optimisation is employed to decide the hyper‐ and architectural parameters of the CNN model. The proposed CNN architecture classifies the chest X‐ray images of COVID‐19 patients into four severity stages, that is, mild, moderate, severe and critical. The proposed CNN model has 16 weighted layers (one input, three convolutions, three ReLU, one normalisation, three max pooling, two fully connected, one dropout, one softmax and one classification layers) as can be seen in Figure 2. Now that this model aims to classify an image into four classes, the output layer has four neurons. The last FC layer, which is a four‐dimensional feature vector, is given as an input to the softmax classifier, which makes the final prediction about the lung severity stage. Detailed information about the layer features such as the size of input images, layer activations and the total number of learnable parameters of each layer is given in Table 2.

**TABLE 2:** The proposed CNN architecture details

	**CNN layer**	**Layer type**	**Layer activations**	**Learnable parameters**	**Total learnables**
1	227 × 227 × 3 input layer	Input	227 × 227 × 3	**—**	0
2	128 6 × 6 × 3 convolutions with stride [4 4] and padding [0 0 0 0]	Convolutional	56 × 56 × 128	Weights: 6 × 6 × 3 × 128 Bias: 1 × 1 × 128	13,952
3	ReLU‐1	ReLU	56 × 56 × 128	**—**	0
4	Cross channel normalisation	Normalisation	56 × 56 × 128	**—**	0
5	2 × 2 max pooling with stride [2 2] and padding [0 0 0 0]	Max pooling	28 × 28 × 128	**—**	0
6	96 6 × 6 × 128 convolutions with stride [1 1] and padding [2 2 2 2]	Convolutional	27 × 27 × 96	Weights: 6 × 6 × 128 × 96 Bias: 1 × 1 × 96	46,752
7	ReLU‐2	ReLU	27 × 27 × 96	**—**	0
8	2 × 2 max pooling with stride [2 2] and padding [0 0 0 0]	Max pooling	13 × 13 × 96	**—**	0
9	96 2 × 2 × 96 convolutions with stride [1 1] and padding [2 2 2 2]	Convolutional	16 × 16 × 96	Weights: 2 × 2 × 96 × 96 Bias: 1 × 1 × 96	36,864
10	ReLU‐3	ReLU	16 × 16 × 96	**—**	0
11	2 × 2 max pooling with stride [2 2] and padding [0 0 0 0]	Max pooling	8 × 8 × 96	**—**	0
12	512 fully connected (FC) layer	FC	1 × 1 × 512	Weights: 512 × 6144 Bias: 512 × 1	3,146,240
13	30% dropout	Dropout	1 × 1 × 512	**—**	0
14	Four FC layer	FC	1 × 1 x 4	Weights: 4 × 512 Bias: 4 × 1	2052
15	Softmax	Softmax	1 × 1 × 4	**—**	0
**16**	**Output with ‘**Mild’**, ‘**Moderate**’, ‘**Severe**’ and ‘**Critical**’ outputs**	**Classification**	—	—	**0**

## 4 EXPERIMENTAL RESULTS

### 4.1 CNN hyper‐parameters optimisation

The parameter of a deep CNN whose value must be set before the learning process is initiated is called a hyper‐parameter. Different methods have been widely used for hyper‐parameter setting, which is often a time‐consuming process. Hyper‐parameter tuning is simply the process of selecting a set of hyper‐parameters for a learning framework. With the deepening of the architectures developed to achieve more successful results and the higher quality of the images used, more computational costs arise. Both the reduction of these calculation costs and the achievement of successful results depend on the use of powerful hardware and optimising the hyper‐parameters of the established network.

It is important to take into account not only the number of hyper‐parameters but also the hyper‐parameters value range while choosing the CNN hyper‐parameters optimisation method. However, when the number of hyper‐parameters to be optimised increases in addition to the specified wide parameter value ranges, the required computation time becomes excessively high, which makes the optimisation process unpractical from the computation point of view. The grid search optimisation method is an efficient alternative for hyper‐parameter optimisations of CNN's when the value range is a small search space. Grid search, which is typically an exhaustive searching through a manually specified range of hyper‐parameter space of a learning framework, has been recently adopted in machine learning as a hyper‐parameter tuning tool. The grid search aims to select the best combination of which the network is trained in all specified range combinations.

In this study, the CNN hyper‐parameters needed to be optimised are grouped in two categories: Architectural hyper‐parameters (which are number of convolutional and max pooling layers (CML), number of FC layers (FCL), number of filters (NF), filter sizes (FS), activation function (AF)) and fine adjustment hyper‐parameters (which are ℓ_2_ regularisation (L2R), momentum (M), mini‐batch size (MBS) and learning rate (LR)). The NF, FS, AF, FCL, CML form the most effective architectural hyper‐parameters, whereas L2R, M, MBS and LR form the most effective fine adjustment hyper‐parameters of CNN models in terms of classification accuracy and efficiency. Once the architectural hyper‐parameters are tuned, fine adjustment hyper‐parameters are tuned based on the architectural hyper‐parameters. Algorithm 1 demonstrates the grid search algorithm adopted to optimise the architectural hyper‐parameters of the proposed CNN models.

Algorithm 1 **Grid search algorithm to optimise the architectural hyper‐parameters**

**Table:** 

**1**.	**Start**
2.	Initialise CML, FCL, NF, FS, AF with default values
**3**.	**for** CML in [1, 2, 3, 4]
4.	**for** FCL in [1, 2, 3, 4]
5.	**for** NF in [16, 24, 32, 48, 64, 96, 128]
6.	**for** FS in [3, 4, 5, 6, 7]
7.	**for** AF in [ELU, SELU, ReLU, Leaky ReLU]
8.	model = CNN_train (train, CML, FCL, NF, FS, AF)
9.	score = CNN_predict (test, model)
10.	cv_list.insert (score)
11.	scores_list.insert (mean(cv_list), CML, FCL, NF, FS, AF)
**12**.	**return** max (scores_list)

**CML**: Number of convolutional and max pooling layers, **FCL**: Number of FC layers, **NF**: Number of filters, **FS**: Filter sizes, **AF**: Activation function

Fine adjustment hyper‐parameters are tuned based on the architectural hyper‐parameters, which have been determined at the first step. At the second step, fine adjustment hyper‐parameters are tuned. Algorithm 2 demonstrates the grid search algorithm adopted to optimise the fine adjustment hyper‐parameters of the proposed CNN models.

Algorithm 2 **Grid search algorithm to optimises the fine adjustment hyper‐parameters**

**Table:** 

1.	**Start**
2.	Initialise L2R, M, MBS, LR with default values
3.	**for** L2R in [0.0001, 0.0005, 0.001, 0.005]
4.	**for** M in [0.80, 0.85, 0.90, 0.95]
5.	**for** MBS in [4, 84, 16, 32, 64]
6.	**for** LR in [0.0001, 0.0005, 0.001, 0.005]
7.	model = CNN_train (train, L2R, M, MBS, LR)
8.	score = CNN_predict (test, model)
9.	cv_list.insert (score)
10.	scores_list.insert (mean(cv_list), L2R, M, MBS, LR)
11.	**return** max (scores_list)

**L2R**: ℓ_2_ regularisation, **M**: Momentum, **MBS**: Mini‐batch size, **LR**: Learning rate

Table 3 demonstrates optimum hyper‐parameters achieved by the grid search optimisation algorithm.

**TABLE 3:** Optimum hyper‐parameters results achieved by grid search

**Parameters**	**Range of parameters**	**Optimum**
Number of convolution and max pooling layers	[1, 2, 3, 4]	3
Number of FC layers	[1, 2, 3, 4]	2
Number of filters	[16, 24, 32, 48, 64, 96, 128]	128, 96, 96
Filter size	[3, 4, 5, 6, 7]	6, 6, 2
Activation function	[ELU, SELU, ReLU, Leaky ReLU]	ReLU
Mini‐batch size	[4, 8, 16, 32, 64]	64
Momentum	[0.80, 0.85, 0.9, 0.95]	0.9
Learning rate	[0.0001, 0.0005, 0.001, 0.005]	0.0001
ℓ_2_regularisation	[0.0001, 0.0005, 0.001, 0.005]	0.001

**FIGURE 3:**
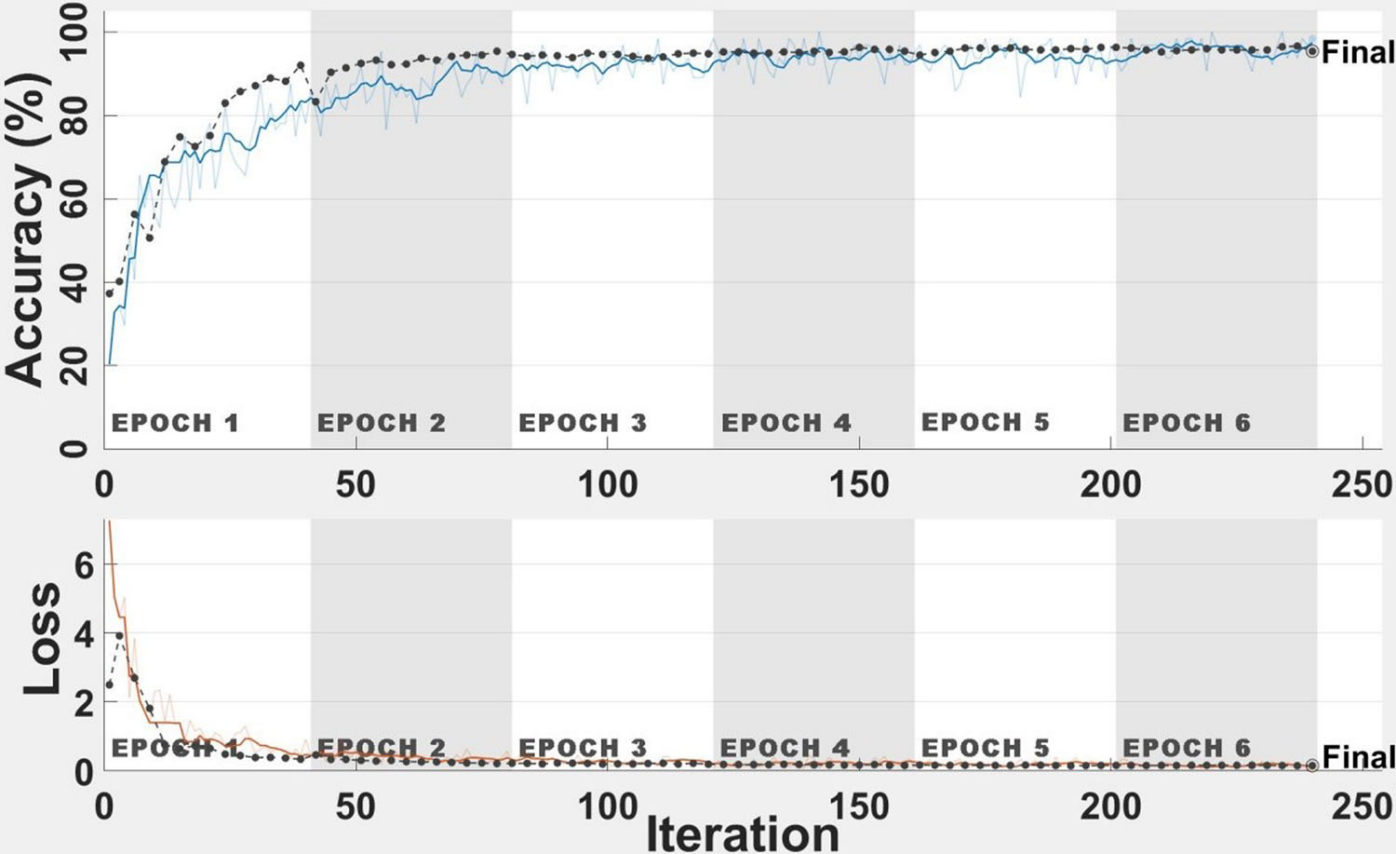
Accuracy and loss curves

**FIGURE 4:**
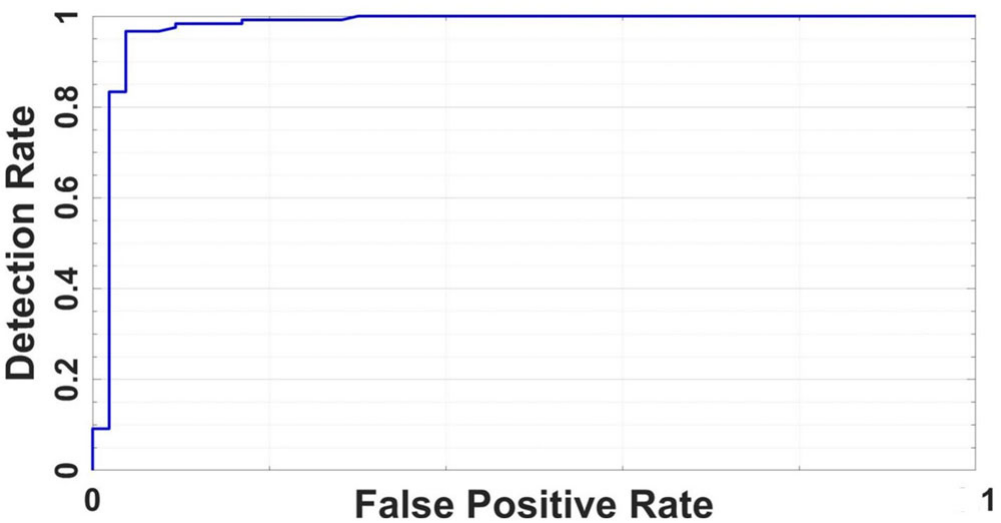
Receiver operating characteristic curve

The classification study is performed using the CNN architecture and tuned hyper‐parameters above, and the results are added in this paper. Figure 3 is the accuracy and loss plot of the proposed CNN model. Average classification accuracy of 95.52% is achieved after 240 iterations using the proposed model. The average AUC value of the ROC curve is 0.9873 as shown in Figure 4. These results show the ability of the proposed CNN model for COVID‐19 disease lung severity staging.

### 4.2 Performance evaluation

The accuracy and validity of image classification methods must be proven by evaluations such as performance evaluation metrics. There are a lot of well‐known and commonly used performance evaluation metrics for image classification problems in the literature. These metrics are derived from the confusion matrix, which is a table that is used to describe the performance of classification models. Accuracy, specificity, sensitivity and precision are considered the most popular performance evaluation metrics. The performance evaluation of the models in this paper is made using the aforementioned metrics in addition to the area of ROC known as AUC of ROC curve value. Corresponding formulas regarding each of these metrics can be seen in Equation 1, where TP, TN, FP and FN are true positive, true negative, false positive and false negative, respectively:

(1) $$\begin{array}{c} \mathrm{Accuracy}=\frac{\mathrm{TP}+\mathrm{TN}}{\mathrm{TP}+\mathrm{TN}+\mathrm{FP}+\mathrm{FN}}\\ \mathrm{Specificity}=\frac{\mathrm{TN}}{\mathrm{TN}+\mathrm{FP}}\\ \mathrm{Sensitivity}=\frac{\mathrm{TP}}{\mathrm{TP}+\mathrm{FN}}\\ \mathrm{Precision}=\frac{\mathrm{TP}}{\mathrm{TP}+\mathrm{FP}} \end{array}$$

**FIGURE 5:**
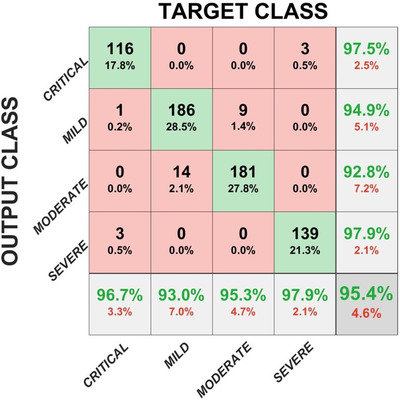
Confusion matrix

**FIGURE 6:**
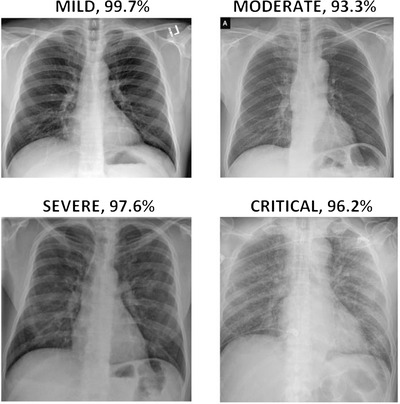
Classification results and the predicted probabilities of four test images

Please see Figure 5 for confusion matrix and Table 4 for accuracy metrics in terms of TP, TN, FP, FN, accuracy, specificity, sensitivity and precision. As shown in Table 4, accuracy of 96.32% is achieved to classify mild stage, 96.47% for moderate stage, 99.08% for severe stage and 98.93% for clinical stage of COVID‐19 disease lung severity classification. Figure 6 shows classification results and the predicted probabilities of four test images.

**TABLE 4:** Accuracy metrics in terms of true positive (TP), true negative (TN), false positive (FP), false negative (FN), accuracy, specificity, sensitivity and precision

**Metrics**										
**Architecture**	**Classes**	**TP**	**TN**	**FP**	**FN**	**Accuracy**	**Specificity**	**Sensitivity**	**Precision**	**Total**
***Proposed CNN Architecture***	**Mild**	186	442	10	14	96.32%	0.978	**0.930**	0.949	200
	**Moderate**	181	448	14	9	96.47%	0.970	0.953	0.928	190
	**Severe**	139	507	3	3	**99.08%**	0.994	**0.979**	**0.979**	142
	**Critical**	116	529	3	4	98.93%	**0.994**	0.967	0.967	120

The performance of the proposed model for COVID‐19 severity classification is evaluated using the five‐fold cross‐validation procedure for COVID‐19 disease severity assessment. Dataset is split into five‐fold and for each fold, four out‐of‐fold observations are used to train the classifier and the remaining fold is used to test the trained classifier. The experiments are repeated five times. Classification performance for the task is evaluated for each fold, and the average classification performance of the model is calculated. Performance metrics are calculated using the results from the confusion matrix, and the corresponding results are shown in Table 5. The average accuracy and AUC scores over all folds are adopted as the final result.

**TABLE 5:** Average classification performance and accuracy metrics for each fold

		**Fold‐1**	**Fold‐2**	**Fold‐3**	**Fold‐4**	**Fold‐5**	**Average**
Performance Metrics (%)	*Sensitivity*	95.72	96.88	94.86	97.14	95.51	96.02
	*Specificity*	98.40	99.14	98.86	98.03	97.71	98.43
	*Precision*	95.58	99.84	98.85	98.03	97.72	98.00
	***Accuracy***	95.40	96.01	94.79	96.93	94.63	**95.52**
	*Area under the curve* (***AUC)***	0.9976	0.9917	0.9788	0.9945	0.9738	**0.9873**

## 5 DISCUSSION

In this artificial intelligence (AI)‐assisted deep learning‐based study, a novel CNN method is developed and validated to classify the COVID‐19 infected patients according to their severity levels as mild versus moderate versus severe versus critical. Experimental results suggest that the proposed method can accurately predict disease severity in COVID‐19 patients using X‐ray imaging, offering promise for clinical diagnosis and early treatment. Many other authors have also detected the COVID‐19 disease through CNN recently. However, the number of images used to train and test the CNN in this proposed study is higher than the number of images used in the previously published studies. For a valid machine learning study, there must be a high number of images to train the neural network. In addition to this, a separate testing dataset has to be used along with training and validating datasets to test the performance of the trained CNN on predicting unseen samples for detecting COVID‐19. Therefore, a test dataset having 652 images is randomly assigned and separated along with a training dataset having 652 images and a validation dataset having 1956 images to comprehensively test the performance of trained CNN on predicting samples. Experimental results on the sufficiently large number of chest X‐ray images demonstrate the effectiveness of the CNN model produced with the proposed framework.

The method proposed in this paper has other aspects that are superior to similar methods in the literature. To compare the proposed model with other deep learning models and to show the worth of the work, the same experiments are conducted using ResNet‐101, ResNet‐34, AlexNet, Visual Group Geometry‐16 (VGG16), and the results are compared with the results of the proposed model. Compared with other supervised AI‐assisted predictive models, the proposed model outperforms other deep learning models in terms of accuracy and AUC as shown in Table 6. The overall accuracy found using the VGG16 model, which is the closest one to the proposed model in terms of overall accuracy, is 88.34%, which is quite less than the 95.52% obtained by the proposed CNN model.

**TABLE 6:** Performance metrics of disease severity classification results under different network models

	**ResNet‐101**	**ResNet‐34**	**AlexNet**	**VGG16**	**Proposed model**
Accuracy (%)	79.87	81.85	75.76	88.34	**95.52**
AUC	0.8109	0.8911	0.749	0.8742	**0.9873**
Elapsed time (s)	1078	875	1287	1707	**720**

The proposed study also differs from other methods in that the CNN hyper‐parameters are automatically optimised. Optimising the hyper‐parameters based on the input images is very important for computation efficiency. In this study, the important hyper‐parameters are automatically tuned using grid search. It is worth to note that observing the trends of grid search is more important than focusing on the only best performing result while deciding the configuration values. That is why reviewing all grid search results should be adopted. A general opinion should be created rather than trying to looking for the best fit values for hyper‐parameters. This should be followed by observing fixed intervals and the relationships or trends among the parameters, which will result in a subset of the dataset that saves time in the long‐timed training process. Elapsed time to train the different CNN models and the proposed CNN model is also shown in Table 6. The computation efficiency of the proposed method can be clearly seen in Table 6. The proposed model needs 720 s for training while maintaining high accuracy. It is seen that the closest model to the proposed model in terms of the time spent on training the model is the ResNet‐34 model with 875 s.

Another method rather than the AI‐assisted methods in the literature for predicting the severity classification of COVID‐19 disease is the radiological severity scoring method, which is based on the radiologist's manual assigning a severity score to each image. This is a subjective method that relies on the experience and expertise of the radiologist. The proposed deep learning method is also superior to this radiological severity scoring method, as the deep learning model eliminates the time‐consuming and subjective severity assignment. Compared with other subjective radiological severity scoring methods, the proposed method not only saves radiologists and clinicians a great deal of time by eliminating the huge workload of the manual annotating the lesion, but it also enables radiologists and clinicians to handle effectively and quickly in dealing with pandemic emergencies.

### 5.1 Comparison of the proposed method with state‐of‐the‐art methods

As stated previously in the related work section, many of the deep learning‐based COVID‐19 studies have been reported since the outbreak over the past year. Most of them are about COVID‐19 disease detection, with only some specifically designed for severity classification (assessment). Among the COVID‐19 disease severity assessment studies using deep learning methods, the disease severity has been divided into severe and non‐severe, reducing the severity assessment into a binary classification. For example, He et al. [12] proposed an automated learning approach to classify COVID‐19 patients into severe and non‐severe groups. An accuracy of 98.5% was obtained using 666 chest CT images, which is not high enough for a valid learning model. Another binary classification of COVID‐19 disease severity was by Tang et al. [16] who suggested a machine learning method based on quantitative features of CT lungs to estimate COVID‐19 disease severity. They achieved 87.5% accuracy for binary classification of COVID‐19 patients. Xiao et al. [17] aimed to build an artificial‐intelligence‐assisted tool to estimate COVID‐19 patients as severe or non‐severe. They achieved an overall accuracy of 81.9% using chest CT images of 408 COVID‐19 patients. Another study was by Yu et al. [18] who exploited a pre‐trained deep neural network and achieved 95.34% accuracy using 729 CT images of COVID‐19 patients to classify disease severity as severe and non‐severe. Carvalho et al. [19] made use of ANN computer‐aided diagnosis to divide COVID‐19 patients into mild, moderate and severe cases. They obtained 82% overall accuracy using 229 CT scans of COVID‐19 patients. Other researchers who realised COVID‐19 disease severity as mild versus moderate versus severe were Zhang et al. [20] who proposed a previously developed deep learning method for COVID‐19 severity classification. They obtained an overall accuracy of 91.6% using 661 CT images.

In summary, most of the deep learning‐based COVID‐19 disease studies belong to disease detection. The remaining related literature shows that the COVID‐19 disease severity assessment is generally about binary classification (severe vs. non‐severe) rather than multi‐classification of COVID‐19 disease severity. It is also quite obvious that the number of images is not high enough to comprehensively train the deep learning models and prevent a biased dataset assignment effect on the CNN to ensure comprehensively testing the CNN model.

Considering the literature carefully, to the best of the author's knowledge, the main advantages and contributions of the proposed approach in this paper are as follows:

1. Many other authors have also detected the COVID‐19 disease through CNN recently; however, the number of images used to train and test the CNN in this proposed study is higher than the number of images used in previous studies. In addition, the proposed study differs from other disease detection studies as it not only makes COVID‐19 disease detection but also achieves the disease severity assessment.
2. This is the first COVID‐19 disease severity assessment study with four stages (mild vs. moderate vs. severe vs. critical) from chest X‐ray images using CNN whose almost all hyper‐parameters are automatically tuned by the grid search optimiser.
3. Thanks to the proposed novel CNN model for COVID‐19 disease severity assessment, multi‐classification of patients’ severity can be achieved with a high classification result such as 95.52%.
4. Comparing with COVID‐19 disease assessment studies based on deep learning approaches, this study eliminates the big workload of segmenting the lesions, which saves time and increases the capacity of clinicians to quickly and effectively handle the COVID‐19 pandemic.
5. On the contrary of time‐consuming and subjective radiological severity scoring methods in which radiologist manually assigns a severity score to each X‐ray image, the proposed fully automatic CNN method is more objective, faster, non‐invasive and does not depend on expertise and experience.

There are several limitations to this study. For instance, the lung disease severity score has been computed with a small group of radiologists (*n* = 2). The consistency of the severity score should be validated with more experienced readers in the future work. Moreover, since this study focuses only on COVID‐19 disease pneumonia and lacks to be applied to assess other pneumonias, the study will be extended to classify other pneumonia severity in the future work. Finally, it will be interesting to construct a cloud‐based publicly available website that uses the proposed system, which can be helpful for researchers to fight and handle the COVID‐19 pandemic.

## 6 CONCLUSION

Now that the radiologists differ in skills and experience, an incorrect staging of the disease severity can lead to more deaths of the COVID‐19 patients. This paper suggests a novel implementation of CNN approach to divide COVID‐19 patients into four severity stages: Mild versus moderate versus severe versus critical with an average accuracy of 95.52%. It is believed that this study has a great potential to lighten the workload of the overloaded frontline radiologists and accelerate the diagnosis, treatment of patients, and thus ease the control of the pandemic.
